# Beneficial effect of adding pentoxifylline to processed semen samples on ICSI outcome in infertile males with mild and moderate asthenozoospermia: A randomized controlled prospective crossover study

**Published:** 2013-11

**Authors:** Medhat Amer, Bahgat Metawae, Hossam Hosny, Ahmad Raef

**Affiliations:** 1 *Department of Andrology and STDs, Kasr Al-Ainy Faculty of Medicine, Cairo University, Cairo, Egypt.*; 2 *IVF Unit, Adam International Hospital, Giza, Egypt*

## Abstract

**Background:** No extensive studies were done that included the use of pentoxifylline or verify its effect on the outcome of ICSI in cases of mild and moderate asthenozoospermia.

**Objective: **The aim of this study was to evaluate the effect of pentoxifylline used in preparation of semen samples which doesn't need motility enhancement prior to ICSI.

**Materials and Methods:** The study was carried on 30 infertile patients where pentoxifylline was used for semen processing (group I), another 30 patients without pentoxifylline (group II) in addition to 60 infertile patients where crossing over of the semen sample was done further subdividing it into 2 subgroups in which the first half of the semen sample was incubated with pentoxifylline (group IIIA) and the second half of the sample without pentoxifylline (group IIIB).

**Results: **The numbers of oocytes injected, numbers of oocytes fertilized, fertilization rate, the total numbers of embryos, numbers of good embryos and the numbers of embryos transferred of group IIIA were found significantly higher than that of Group IIIB (p=0.00). The overall 6 month pregnancy rate of group I was significantly higher than that of group II (73.3% vs. 60% respectively, p=0.04). The abortion rate of (Group I) and that of (Group II) was found non – significantly different (20% vs. 27.8% respectively, p=0.53).

**Conclusion:** Pentoxifylline can be used as a useful compound for improving ICSI outcome in semen samples preparation prior to oocytes injection regardless of the state of sperm motility or the degree of asthenozoospermia.

**Registration ID in Clinical Trials.gov:** NCT01793272

## Introduction

Pentoxifylline (PTX) is a non-specific phosphodiesterase (PDE) inhibitor of the methylxantine group, it inhibits the breakdown of cyclic adenosine monophosphate (cAMP) and it is known that intracellular cAMP concentration plays a central role in sperm motility (1). The molecular basis of cAMP regulation is based on protein phosphorylation, especially for capacitation as (2). 

The typical protocol for PTX use involves a 30 minutes preincubation of prepared sperm with stimulant (PTX at 1-5 mmol/l), sperm is then washed to remove the stimulant and is used immediately for ova fertilization (3). Apart from the effects on sperm motility, Tesarik *et al* reported that PTX augments the acrosome reaction (4). No extensive studies were done that included the use of pentoxifylline or verify its effect on the outcome of ICSI in cases of mild and moderate asthenozoospermia, although extensive studies were done on its effect in cases of severe male factor, severe oligozoospermia or severe asthenozoospermia particularly in IVF cycles. 

Rizk *et al* in a study on IVF cycles found that a significantly higher fertilization rate occurred in the group were oocytes were inseminated with spermatozoa treated with PTX compared with controls (5). Imoedemhe et al showed an increase in sperm motility but a significant decrease in the fertilization rates and embryo development (6). Sohn et al reported that the fertilization rate and clinical pregnancy of PTX group was higher than those of ICSI programs undertaken using sperm not treated with PTX (7).

Takeda *et al* found that fertilization and embryo recovery rates of the PTX group were significantly lower than those of the control group in csases of severe oligozoospermia (8). Therefore, this randomized prospective crossover study was performed to demonstrate, for the first time in literature, the beneficial effect of PTX used in preparation of semen samples that will be used for ICSI in infertile men complaining of mild and moderate asthenozoospermia in comparison to semen samples without PTX preparation on the outcome of ICSI.

## Materials and methods

**Study** **population**

**Figure 1:**
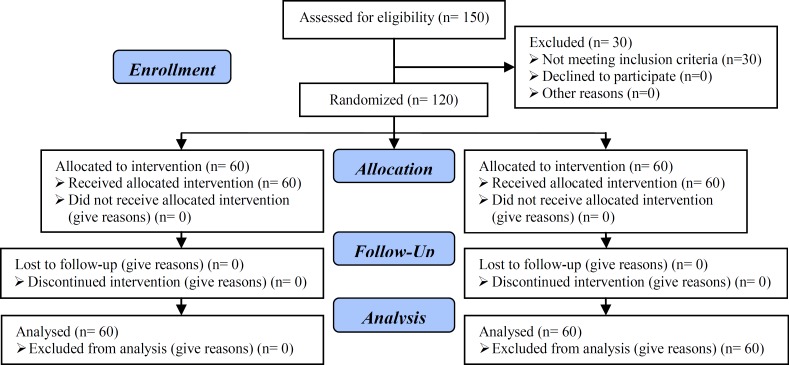
Consort flow diagram

This study recruited 150 subjects, from October 2010 to April 2011, from males attending the Andrology Clinic in a specialized center for assisted reproduction techniques (Adam International Hospital, Giza, Egypt) seeking ICSI for primary or secondary infertility as shown in the consort flow diagram 2010 (Figure 1). All patients were provided with a through history taking, both present and past, general and local genital examination, conventional semen analysis according to WHO 2010 standards, and referral of the patient's wife to the gynecology and obstetrics unit in the same center for through assessment and preparation for ICSI (9).

Any patient with following criteria was excluded from participating in the study: Semen profile showing pyospermia, presence of antisperm antibodies, azoospermia, severe male factor, oligozoospermia or severe asthenozoospermia. Wife’s age >35 years. Low antral count. Presence of any ovarian factor contributing to infertility e.g. PCO. Past history of orchitis. Patients who received empirical treatment for asthenozoospermia e.g. oral PTX, l-carnitine or antioxidants during the past 3-6 month. Diabetics, hypertensives or patients with any other chronic systemic illnesses. 

After excluding 30 subjects due to one or more of the previously mentioned exclusion criteria, 120 males were included in the study with semen showing mild or moderate asthenozoospermia and they were prospectively randomized and divided into 3 groups:

- **Group (I):** consisted of 30 patients where semen sample was incubated with PTX on the day of ova pickup prior to ICSI.
- **Group (II):** consisted of 30 patients where semen sample was processed without using PTX on the day of ova pickup prior to ICSI.
- **Group (III):** consisted of 60 patients where cross over was done as the semen sample obtained from each patient on day of ova pickup was divided equally into two parts, further subdividing these patients into 2 subgroups: Group (IIIA) PTX-treated semen portion and Group (IIIB) non-PTX treated control semen portion and the wife’s ova was divided over these 2 parts for fertilization.

**Study** **design**

The study randomization was performed using computer generated random numbers for each patient presenting at the Andrology Clinic in the hospital and informed consent was obtained from all patients and after receiving a detailed explanation from the physician according to the regulations of the "Andrology Department Ethical Committee, Faculty of Medicine, Cairo University".

**Study** **protocol**

Female partners were prepared by the long controlled ovarian stimulation protocol after performing the general, reproductive and hormonal evaluations of the patient. Cumulus-corona-oocyte complexes were retrieved by vaginal ultrasound-guided puncture of the ovarian follicles performed 35 hours after human chorionic gonadotrophin (HCG, Choriomon, IBSA) administration when the criteria for oocyte maturity were met as stated by Palermo *et al* (10). Two hours after collection, the oocytes were treated by 0.5% hyaluronidase (Hyase, Ferti Pro) for 10 seconds for enzymatic removal of the cumulus oophorus cells using a sterile yellow tip. 

Under stereomicroscopic guidance at x50 magnification, cells of the corona radiata were removed mechanically with aid of 140 um cook stripper (Cook, Australia) for fine denaturation, then the maturity of the oocytes was determined, only oocytes in metaphase ΙΙ were injected as shown by Ola *et al* (11). Pentoxifylline (Trental ®) 100mg/ 5ml ampoules (Sanafi Aventis, Istanbul, Turkey) was used where 20 microns were aspirated by pipette and dissolved in either Earl’s Buffered Solution (EBS, GIBCO®) or Human Tubal Fluid (HTF, Irvine Scientiﬁc®) medium, then solution was incubated with freshly collected semen sample at 37^o^C and was ready for ova fertilization after about 5 minutes. 

The preparation of the holding and injection pipettes, and the injection procedure itself, were described by Al-Hasani *et al* (12). Sixteen to eighteen hours after injection and incubation in 20 ul microdrops of medium under Sage cleavage culture medium and Sage oil as demonstrated by Quinn, for tissue culture at 37^o^C in a humidified atmosphere with 6% CO_2_ and 5% O_2_; the oocytes were examined for the presence of pronuclei and polar bodies as shown by Tesarik and Greco (13, 14). The embryos were monitored exactly 48 hours for 4 cell stage (day 2 transfer), or exactly 72 hours for 7-9 cell stage (day 3 transfer), or blastocyst stage (day 5 transfer). Embryo scoring was based on the following criteria: 

A-Blastomere number (cleavage rate), B-Fragmentation percentage and C-fragmentation pattern. Each item given certain number of points and the cumulative embryo score was determined by (blastomere number points X fragmentation % points± extrapoints). Good embryo took 15-20 points, fair embryos took 10-15 while poor embryos took 0-10 points according to the findings of Alikani *et al* (15). Luteal support was provided with Prontogest (IBSA, Switzerland) until the pregnancy test was performed by measuring the serum HCG concentration on at least two separate occasions, 2 weeks after embryo transfer. Clinical pregnancy was determined by ultrasound screening of the fetal sac, fetal pole and cardiac beat after 3 weeks for patients with positive pregnancy test. Pregnancy was monitored during the following 6 month by the obstetrician and the final outcome was reported to the clinic. 

**Study end-points**

The primary outcome measures were the number of oocytes retrieved, the number of oocytes injected, number of oocytes fertilized, fertilization rate, number of embryos and their quality (G1: good embryos, G2: fair embryos and G3: poor embryos), number of embryos transferred (ET), number of embryo sacs, embryo implantation rate, pregnancy rate and abortion rate for all 3 groups and pregnancy rate and abortion rate for both group I and group II.

**Statistical analysis**

Student T-test, Chi-square test, Mann-Whitney U-test were used for comparing data from all groups. Results were expressed as means, standard deviation (SD). Statistical significance was accepted at a p<0.05.

## Results

Using Mann-Whitney test, the fertilization rate, number of good embryos, number of fair embryos, number of embryos transferred, number of embryos sacs and the implantation rate of group I patients and those of group II patients were found non-significantly different (p=0.935, 0.377, 0.833, 0.755, 0.091 and 0.132 respectively). The numbers of oocytes retrieved, numbers of oocytes injected, numbers of oocytes fertilized, numbers of poor embryos and the total numbers of embryos of group II patients were found higher than that of group I patients and the difference was significant (p=0.005, 0.007, 0.02, 0.004 and 0.028 respectively).

**Figure 2:**
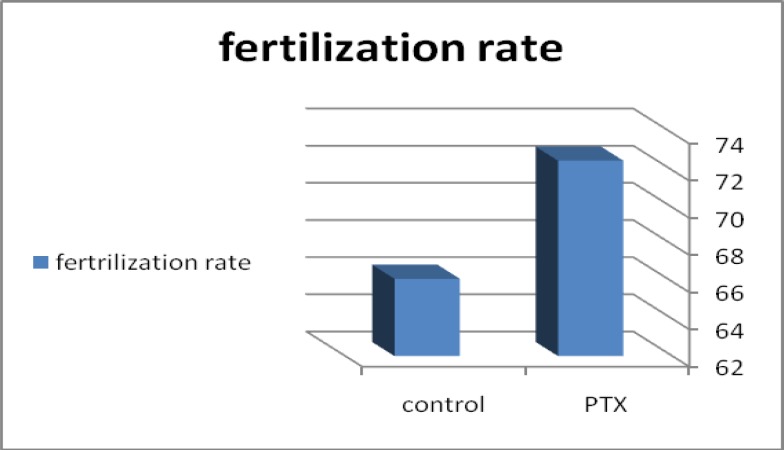
Bar chart comparing fertilization rate between group IIIA (semen treated with PTX, 72.5%) and group IIIB (control semen, 66.1%).

Using Chi-Square test, the pregnancy rates (Table I) and abortion rates of group I was compared to that of group II. The pregnancy rate of group I patients (73%, 22 patients) was found higher than that of group II patients (60%, 18 patients) and the difference was significant. The abortion rate of group I patients (20%, 6 patients) was found lower than that of group II patients (27.8%, 5 patients) but the difference was non-significant. Using Student t-test and paired sample tests (Table II) the numbers of oocytes injected, numbers of oocytes fertilized, fertilization rate (Figure 2), the total numbers of embryos, numbers of good embryos and the numbers of embryos transferred of group IIIA were found higher than that of group IIIB) and the difference was significant. The numbers of fair embryos and the numbers of poor embryos of group IIIA and those of group IIIB were found non-significantly different.

**Table I:** Comparing group I with group II regarding pregnancy rates

**Pregnancy**	**Group**			**p-value** ** sig. (2-tailed)**
	**Group I**	**Group II**	**Total**	
Count	22	18	40	0.0473
% within pregnancy	55.0%	45.0%	100.0%	
% within group	73.3%	60.0%	66.7%	

Using Chi-Square test, the pregnancy rates and abortion rates of group I was compared to that of group II.

**Table II:** Comparing group IIIA with group IIIB

		**N**	**Mean**	**SD**	**SE mean**	**p-value** **sig. (2-tailed)**
Pair 1						0.005
	Oocytes injected PTX	60	7.4333	2.41722	0.31206	
	Oocytes injected	60	7.1500	2.60914	0.33684	
Pair 2						0.002
	Fertilized oocytes PTX	60	5.5167	2.37567	0.30670	
	Fertilized oocytes	60	5.0167	2.76474	0.35693	
Pair 3						0.003
	Fertilization rate PTX	60	72.5183	18.20812	2.35066	
	Fertilization rate	60	66.1600	24.85231	3.20842	
Pair 4						0.000
	Embryo number PTX	60	4.5000	1.91780	0.24759	
	Embryo number	60	3.6167	2.27806	0.29410	
Pair 5						0.000
	G1 embryos PTX	60	2.4167	1.45313	0.18760	
	G1 embryos	60	1.5833	1.51032	0.19498	
Pair 6						0.074
	G2 embryos PTX	60	1.3500	0.79883	0.10313	
	G2 embryos	60	1.1333	0.91070	0.11757	
Pair 7						0.159
	G3 embryos PTX	60	0.7500	0.79458	0.10258	
	G3 embryos	60	0.9167	0.82937	0.10707	
Pair 8						0.000
	ET PTX	60	2.0833	0.71997	0.09295	
	ET	60	0.8333	0.86684	0.11191	

G1: Number of good embryos.

G2: Number of fair embryos.

G3: Number of poor embryos.

ET: Number of embryos transferred.

Using Student *t*- test and paired sample tests, the parameters of group IIIA was compared to the parameters of group IIIB.

## Discussion

In a comparative study, Sohn *et al* evaluated the effect of PTX on the conventional ICSI program undergone in severe asthenozoospermia on a total of 348 cycles of ICSI (7). Fertilization rate of PTX group was higher than those of ICSI programs undertaken using sperm not treated with PTX (70.6% vs. 62.9%, p<0.01) and, ET and clinical pregnancy rates of PTX group were slightly higher (93.1%, 44.2% vs. 90.3%, 36.2%). In a study of ICSI in cases with severe oligozoospermia Takeda *et al* stimulated sperm motility using PTX and found that fertilization and embryo recovery rates of the PTX group were significantly lower than those of the control group (8). However, the pregnancy, implantation, and abortion rates of the PTX group were not significantly different from those of the control group. 

Moreover, no abnormal findings were found in babies subsequently born. They concluded that PTX treatment was effective in cases where sperm sorting was difficult. Our results showed that the fertilization rate, the number of good embryos, the number of fair embryos, the number of embryos transferred, the number of embryo sacs and the implantation rate were not significantly different when comparing group I with group II. On the other hand, the number of poor embryos was significantly higher in group II in absence of PTX addition proving that it has no embryotoxic effect. Moreover, the pregnancy rate was significantly higher in group I than that of group II, whereas the abortion rate was found non-significantly different between the two groups. 

The effect of PTX was more evident when comparing group IIIA with group IIIB, where almost all the outcome parameters of ICSI (the number of oocytes fertilized, the fertilization rate, the total number of embryos, the number of good embryos and the number of embryos transferred) were found significantly higher in group IIIA (semen portion incubated with PTX) then in group IIIB (other semen portion not incubated with PTX). Moreover, the number of poor embryos was found significantly lower in group IIIA than in group IIIB, so once again excluding that PTX has embryotoxic effects. Our results agree with the results of Rizk *et al* and Sohn *et al* as regards the positive effect of PTX on the fertilization rates, embryos quality, the number of embryos transferred and with results of the pregnancy rates (5, 7). 

It also agrees with the findings of Takeda *et al* on the fertilization rate being higher with PTX and that the abortion rate is not significantly different when using it, however our study differs from the latter study in that the pregnancy rate was significantly higher in our study, but their study included a larger number of infertile couples (348) than ours, and they did not do crossing over as in the present study and they worked on a different semen parameter (severe OAT) (8). On the other hand, our results contradicts with the findings of Imoedemhe *et al* which stated that using PTX was associated with decrease in both the fertilization rate and the embryo quality, but their study was on IVF cycles and included only 42 oocytes from 21 women (6). 

We concluded that PTX has a significant positive effect on ICSI outcome in cases of mild and moderate asthenozoospermia as regards the fertilization rate, the embryos quality and the pregnancy rates without any significant embryotoxic effect or significant increase in the abortion rate. Moreover, the results are more solid when using prospective crossing over of the semen sample in each patient i.e. each patient acts as his own control. At last but not least, our study presents a conclusion that PTX can be used as a useful compound in semen samples preparation prior to oocytes injection in ICSI procedure regardless of the state of sperm motility or the degree of asthenozoospermia.

## References

[1] Tash JS, Means AR (1983). Cyclic adenosine 3'5' monophosphate, calcium and protein posphorylation in flagellar motility. Biol Reprod.

[2] Flesch FM, Colenbrander B, Van Golde LMG, Gadella BM ( 1999). Capacitation induces tyrosine phosphorylation of proteins in the boar sperm plasma membranes. Biochem Biophys Res Commun.

[3] Mitchell LM, Peter R. Brinsden (2005). Laboratory techniques: Sperm preparation for assisted conception. Textbook of in vitro fertilization and assisted reproduction the Bourn Hall guide to clinical and laboratory practice.

[4] Tesarik J, Mendoza C, Carreras A (1992). Effects of phosphodiesterase inhibitors caffeine and pentoxifylline on spontaneous and stimulus-induced acrosome reaction in human sperm. Fertil Steril.

[5] Rizk B, Fountain S, Avery S, Palmer C, Blayney M, Macnamee M (1995). Successful use of pentoxifylline in male-factor infertility and previous failure of in vitro fertilization: a prospective randomized study. J Assist Reprod Genet.

[6] Imoedemhe DA, Sigue AB, Pacpaco EL, Olazo AB (1992). The effect of caffeine on the ability of spermatozoa to fertilize mature human oocytes. J Assist Reprod Genet.

[7] Sohn JO, Shin JS, Jeong CJ, Cho YS, Oum KB, Choi DH (2002). Effect of pentoxifylline on the ICSI program udergone in severe asthenozoospermia. Korean J Fertil Steril.

[8] Takeda N, Abe A, Akimoto S, Kudo T, Machiya R, Onuma M (2009). Clinical outcome of sorting and recovery of ultrasmall amounts of live sperm stimulated with pentoxifylline followed by intracytoplasmic sperm injection. J Mammalian Ova Res.

[9] World Health Organization, Department of Reproductive Health (2010). WHO laboratory manual for the examination and processing of human semen.

[10] Palermo GD, Cohen J, Alikani M, Adler A, Rosenwaks Z (1995). Intracytoplasmic sperm injection: a novel treatment for all forms of male factor infertility. Fertil Steril.

[11] Ola B, Afnan M, Sharif K, Papaioannou S, Hammadieh N, Barratt CLR (2001). Should ICSI be the treatment of choice for all cases of in-vitro conception?. Hum Reprod.

[12] Al-Hasani S, Küpker W, Baschat AA, Sturm R, Bauer O, Diedrich C (1995). Mini-swim-up: a new technique of sperm preparation for intracytoplasmic sperm injection. J Assist Reprod Genet.

[13] Quinn P ( 2004). The development and impact of culture media for assisted reproductive technologies. Fertil Steril.

[14] Tesarik J, Greco E (1999). The probability of abnormal preimplantation development can be predicted by a single static observation on pronuclear stage morphology. Hum Reprod.

[15] Alikani M, Calderon G, Tomkin G, Garrisi J, Kokot M, Cohen J (2000). Cleavage anomalies in early human embryos and survival after prolonged culture in-vitro. Hum Reprod.

